# Enhancing composition-based materials property prediction by cross-modal knowledge transfer

**DOI:** 10.1038/s41598-026-53182-3

**Published:** 2026-05-20

**Authors:** Ivan Rubtsov, Ivan Dudakov, Yuri Kuratov, Vadim Korolev

**Affiliations:** 1 AI Center, Lomonosov Moscow State University, Moscow, Russia 119991; 2 MSU Institute for Artificial Intelligence, Lomonosov Moscow State University, Moscow, Russia 119192; 3 Cognitive AI Systems Lab, Moscow, Russia

**Keywords:** chemical language models, transformers, multimodal learning, materials property prediction, Chemistry, Materials science, Mathematics and computing

## Abstract

Crystal graph neural networks are widely applicable in modeling experimentally synthesized compounds and hypothetical materials with unknown synthesizability. In contrast, structure-agnostic predictive algorithms allow exploring previously inaccessible domains of chemical space. Here we present a universal approach for enhancing composition-based materials property prediction by means of cross-modal knowledge transfer. Two formulations are proposed: implicit transfer involves pretraining chemical language models on multimodal embeddings, whereas explicit transfer suggests generating crystal structures and implementing structure-aware predictors. The proposed approaches were benchmarked on LLM4Mat-Bench and MatBench tasks, achieving state-of-the-art performance in 25 out of 32 cases. In addition, we demonstrated how another modeling aspect of chemical language models—interpretability—benefits from applying a game-theoretic approach, which is able to incorporate high-order feature interactions.

## Introduction

The duality between material’s constituent parts and its properties, metaphorically referred to as the incarnation of the philosophical duality between body and soul^1^, is increasingly resolved by means of machine learning approaches^2^. Task specificity determines the granularity of materials representation at which prediction model operates. High-throughput computational screening across synthetically accessible compounds is typically carried out using structure-aware models that take into account the crystallographic data. Accuracy of graph neural networks (GNNs) utilized in materials science^3^ has been consistently improved by introducing the convolution operation on crystal graphs^4^, learnable bond and global-state embeddings^5^, many-body interactions^6^, and neighbor equalization^7^. Recently, further performance gains have been achieved with multimodal architectures, which incorporate data beyond the spatial arrangement of atoms^8^. Considering the immense size of chemical space^9^, a much more ambitious strategy is exploring compounds that were not studied experimentally before. However, approaches that explore new chemical domains by generating crystal structures often require significantly more computational resources and introduce more variables than approaches based solely on generating new chemical compositions. Furthermore, transfer from stoichiometric composition to crystalline phase, i.e., crystal structure prediction (CSP)^10^, is a notorious computational task impeding the use of structure-aware models in exploring unknown chemical systems. Consequently, there is a critical need for predictive models that operate on chemical formulas yet achieve accuracy comparable to that of structural models. Driven by this need, composition-based models have been extensively developed as well^11^, starting with classical ML algorithms trained on hand-crafted features^12,13^; the descriptors constructed as analytical expressions deserve special attention^14,15^. Lately, deep learning approaches have shifted the focus from manual feature engineering to the development of universal representations that capture domain knowledge inherently. A pioneering model of this kind, ElemNet^16^, was based on a 17-layered fully-connected architecture, whereas the input vectors consisted of element fractions. Its successors^17–20^ have been advanced by incorporating pretrained element embeddings^21^ and attention mechanisms^22,23^ into training. Diverse pretraining strategies, including self-supervised learning, fingerprint learning, and multimodal learning, have been applied^24^ to improve the efficacy of the Representation Learning from Stoichiometry (Roost) framework^17^ on downstream tasks. One more step in developing accurate models that are based on composition but retain awareness of structural constraints has been made by Na^25^, who proposed the cross-modal transfer learning approach for modeling experimental properties.

**Figure 1:**
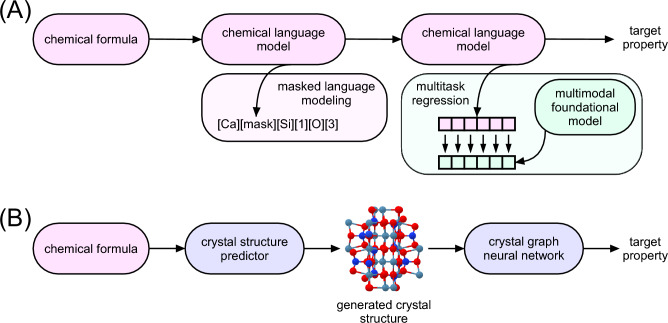
A conceptual scheme of cross-modal knowledge transfer formulations. **A** Implicit knowledge transfer pipeline includes two pretraining phases: masked language modeling (on chemical symbols and stoichiometric coefficients) and multitask regression on multimodal embeddings produced within the foundational model (MultiMat). **B** Explicit knowledge transfer pipeline involves crystal structure prediction in a high-throughput manner and materials property prediction using structure-aware model.

The aforementioned deep learning approaches incorporate chemical composition as a set of elemental or atom-wise attributes, whereas the advent of chemical language models (CLMs) reframes the composition-based property prediction as a sequence modeling task. The models originally trained via masked language modeling (MLM) on materials science abstracts^26^ and crystal text descriptions^27^ have been finetuned and evaluated in reproducing diverse quantities^28^. Here, we take a next step in developing CLMs aimed at composition-based property prediction. In contrast to the previously mentioned MatBERT^26^ and LLM-Prop^27^ models, multiple modalities beyond natural language were incorporated into pretraining (Fig. 1A). Crucially, the predictive accuracy of any ML algorithm is bounded by the representation’s ability to encode the necessary domain knowledge^25^. Therefore, after the MLM stage, embeddings from CLMs were aligned to those of the foundation model recently presented under the framework of multimodal learning for materials^8^ (MultiMat). We utilized the crystal structure encoder that was contrastively pretrained on four materials modalities: crystal structure, density of electronic states, charge density, and textual description. This approach can be characterized as implicit cross-modal Knowledge Transfer (imKT) since the considered property predictors, CLMs, directly operate on modality of interest, i.e., chemical composition. Alternatively, materials property prediction was explicitly transferred (exKT) from the compositional to structural domain with the large language model—CrystaLLM^29^—served as a crystal structure predictor, followed by GNN finetuned on the generated crystals (Fig. 1B). The rest of the paper is devoted to the demonstration of how cross-modal knowledge transfer affects predictive performance, taking into account the current state-of-the-art (SOTA) algorithms. In addition, explainability analysis of CLM outputs is presented, considering high-order token interactions. The main contributions of this work are as follows. First, we introduce a universal cross-modal knowledge transfer framework for composition-based materials property prediction and formulate it in two complementary ways: implicit transfer (imKT), based on aligning CLMs with multimodal materials representations, and explicit transfer (exKT), based on structure generation followed by structure-aware prediction. Second, in contrast to prior CLM-based approaches that rely primarily on language pretraining, we incorporate multimodal materials knowledge beyond text, including information related to crystal structure and other structural modalities, into the representation learning stage. Third, we provide a unified evaluation across LLM4Mat-Bench and MatBench tasks, showing that the proposed approach improves predictive performance in a broad range of endpoints. Finally, we demonstrate that the proposed framework also supports model interpretation by combining CLMs with SHAP-IQ analysis of high-order token interactions.

## Results

Table 1 presents a comparison of our best-performing models with SOTA algorithms available in the literature; a comprehensive picture of how accurately all models perform is provided in Supplementary Tables S1–S5. Considering 20 tasks from the JARVIS-DFT dataset (LLM4Mat-Bench^28^), substantial improvement (from 4.5 to 39.6%) in mean absolute error (MAE) was achieved in 18 cases; on average, the MAE decreased by 15.7%. Knowledge transfer failed to reduce MAE for two tasks: energy above the convex hull and maximal piezoelectric strain coefficient. Prediction of the former property is expectedly complicated by introducing stability-aware weights in the MLM loss function (see details in Supporting Information, Methods section), so CLMs pretrained on multimodal embeddings surpassed models trained via the two-stage procedure (Supplementary Table S6). By applying the imKT approach, we also significantly improved performance in predicting four band-gap-related tasks from the SNUMAT dataset (LLM4Mat-Bench); on average, the MAE decreased by 15.2%.

LLM4Mat-Bench is the largest benchmark for evaluating performance of CLMs in predicting properties of crystalline materials, lacking the assessment of composition-based predictors based on other neural network architectures. To fill this gap, we utilized another well-established suite of predictive tasks, MatBench^30^ (Table 1, Supplementary Tables S4 and S5). The imKT models achieved SOTA results on only three out of eight considered tasks; there is no dominating architecture that provides the best performance on most tasks, as opposed to the LLM4Mat-Bench datasets. An integrative comparison across datasets was done by calculating the weighted average of MAD:MAE ratio, where MAD stands for the mean absolute deviation. The best value of 8.25 was achieved by CrabNet, whereas the top-two value of 8.11 corresponds to imKT@ModernBERT (Supplementary Table S5). Excellent performance of this imKT model relates to advances in the BERT architecture (ModernBERT outperforms BERT and RoFormer, Supplementary Tables S1–S5) and to the cross-modal knowledge transfer: multimodal learning as a pretraining task yields accuracy on par with the two-step procedure (Supplementary Tables S6–S10). To sum up, the imKT technique ensures the highest in-class, i.e., across CLMs, performance (according to LLM4Mat-Bench results), and approaching accuracy of the best-performing architecture (i.e., CrabNet), as it follows from the MatBench results.

Analyzing Supplementary Tables S1–S5 reveals that the imKT models surpass the exKT models across all tasks, lowering MAE by 7.4% on average. We assume that including structural information in the prediction pipeline does not improve accuracy because of two reasons. First, DFT-computed benchmarks contain a substantial fraction of metastable compounds, which are not a target of CSP. For instance, 37% of the JARVIS-DFT compounds have an energy above the convex hull exceeding 0.1 eV/atom, and 6% are highly unstable (${E}_{hull}$ > 1.0 eV/atom). Second, the applied CSP approach has limited ability to output the most stable polymorphs: considering the JARVIS-DFT compounds with ${E}_{hull}$ < 0.01 eV/atom, only a minor portion (11.7%) of the generated crystal structures suites the original ones, in accordance with the structure matching procedure implemented in the pymatgen^31^. Whereas the former issue is inherent to the task, the latter can be addressed by further developing high-throughput CSP algorithms. It should be also noted that training and inference of GPT-like and GNN models (used for generating crystal structures and reproducing target quantities, respectively) may result in substantial computational demands, which are another reason to prioritize CLMs as composition-based property predictors. A quantitative inference-time analysis confirms this practical limitation of the exKT pipeline. All inference measurements were performed with batch size 1 on an NVIDIA A100 GPU. The imKT@ModernBERT model requires 15.07 ms per composition on average (about 15.1 s per 1,000 predictions), whereas crystal-structure generation with CrystaLLM requires 670.09 ms per composition; the subsequent GNN inference is comparatively inexpensive, amounting to 2.79 ms for CGCNN, 13.68 ms for MEGNet, and 3.51 ms for CartNet. Notably, the inference time of the discriminative predictors exhibits only a weak dependence on the number of unique elements in the composition: imKT@ModernBERT remains close to 15 ms across all considered groups, and the three GNN architectures vary only modestly within architecture-specific ranges. In contrast, the generation time of CrystaLLM grows rapidly with compositional complexity, increasing from 235.30 ms for one-element compositions to 1528.43 ms for five-element compositions, with intermediate values of 397.23, 642.88, and 1182.37 ms for two-, three-, and four-element systems, respectively. Thus, the computational bottleneck of the exKT pipeline is the structure-generation stage rather than the downstream graph-based prediction itself. As a result, the total exKT inference cost exceeds that of the CLM-based predictor by approximately 45 times. Nevertheless, we consider exKT as a promising technique, taking into account its current performance: CLMs presented in LLM4Mat-Bench were outperformed in 22 out of 24 cases, summarizing the JARVIS-DFT and SNUMAT tasks (Supplementary Tables S1–S3).

**Figure 2:**
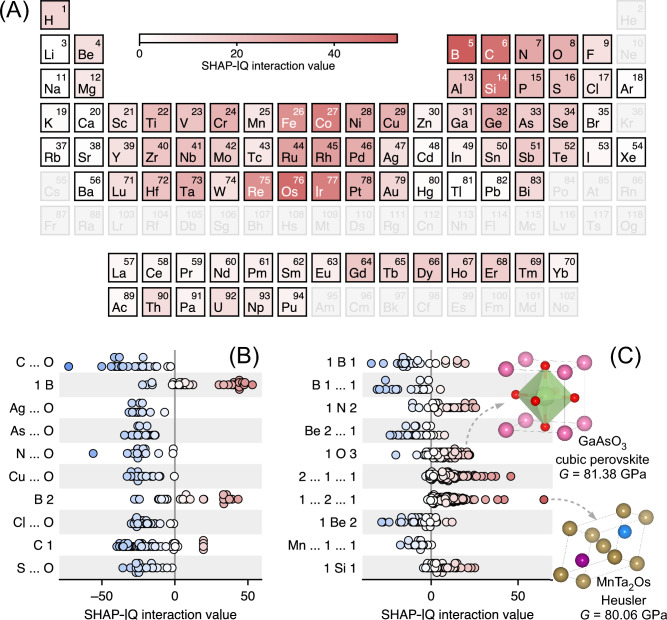
Explainability analysis of chemical language model predicting shear modulus. **A** Element-wise importance scores computed as averaged SHAPley Interaction Quantification (SHAP-IQ) values. Most influential **B** two- and **C** three-token combinations, according to the averaged SHAP-IQ values. Two crystal structures from the JARVIS-DFT dataset are depicted to outline most common structural prototypes; the corresponding DFT-computed shear moduli are provided as well.

Neural networks for materials property prediction are typically utilized as “black-boxes”, missing understanding of their internal machinery. A few exceptions among composition-based models are transformers with attention attributes^18,20^, which are debatably related to feature importances^32^. In this context, we applied a post hoc explainability technique that includes any-order feature interactions to gain insights into structure-property relationships, going beyond element-wise contributions. Specifically, the outputs of the imKT@ModernBERT model for predicting shear modulus (JARVIS-DFT dataset, LLM4Mat-Bench) were processed using the SHAPley Interaction Quantification^33^ (SHAP-IQ) approach. As a starting point for analysis, the averaged elemental contributions are provided in Fig. 2A. Expectedly, the highest impact on the shear modulus value is exerted by the presence of some platinum group metals and non-metals forming (ultra)hard materials, e.g., borides, carbides, and silicides. Considering the most influential two-token combinations (Fig. 2B), we conclude that oxygen-containing groups reduce the target quantity. In contrast, boron-containing sequences increase the shear modulus value in most cases, though further insights can hardly be gained. Three-token combinations are most informative features, which extend our understanding beyond a compositional aspect (Fig. 2C). In particular, the “1 O 3” sequence relates to the cubic perovskite prototype (CaTiO${}_{3}$, E2${}_{1}$ *Strukturbericht* designation, $Pm\overline{3}m$ space group) in 38% of cases, as it follows from analysis of the corresponding JARVIS-DFT crystal structures. The Heusler structure (AlCu${}_{2}$Mn, L2${}_{1}$ *Strukturbericht* designation, $Fm\overline{3}m$ space group) was identified for a noticeable part of “2 … 1 … 1” and “1 … 2 … 1” sequences (41% and 35% of cases, respectively). However, there are no preferable prototypes for other three-token sequences. These findings represent an early example of interpreting chemical compositions through the crystallographic prototype analysis and advanced game-theoretic approach (i.e., SHAP-IQ), which is a promising combination for enhancing CLM explainability.

**Table 1:** Predictive performance comparison of transfer knowledge models with existing architectures. Three separated groups of tasks correspond to JARVIS-DFT, SNUMAT (both are the LLM4Mat-Bench datasets), and MatBench benchmarks. Mean absolute error (MAE) values are provided for the models demonstrating state-of-the-art (SOTA) performance.

predictive task	SOTA existing architecture	SOTA existing MAE $\downarrow$	performance boost $\uparrow$	SOTA presented MAE $\downarrow$	SOTA presented architecture
FEPA	MatBERT-109M	0.126	+8.8%	0.11488 ± 0.00018	imKT@ModernBERT
Band gap (OPT)	MatBERT-109M	0.235	+15.5%	0.1985 ± 0.0019	imKT@BERT
Total energy	MatBERT-109M	0.194	+39.6%	0.1172 ± 0.0005	imKT@ModernBERT
Ehull	MatBERT-109M	0.096	–	0.1031 ± 0.0009	imKT@RoFormer
Band gap (MBJ)	MatBERT-109M	0.491	+23.2%	0.3773 ± 0.0030	imKT@ModernBERT
Kv	MatBERT-109M	18.498	+11.6%	16.35 ± 0.24	imKT@ModernBERT
Gv	MatBERT-109M	14.241	+10.4%	12.76 ± 0.05	imKT@ModernBERT
SLME	MatBERT-109M	5.851	+16.1%	4.911 ± 0.010	imKT@ModernBERT
Spillage	MatBERT-109M	0.409	+15.4%	0.3462 ± 0.0029	imKT@ModernBERT
${\epsilon }_{x}$ (OPT)	MatBERT-109M	32.661	+25.5%	24.32 ± 0.06	imKT@ModernBERT
${\epsilon }$	Gemma2-9b-it:5S	28.228	+5.8%	26.6 ± 0.4	imKT@RoFormer
Max. piezo. (${d}_{ij}$)	Gemma2-9b-it:5S	7.973	–	9.67 ± 0.10	imKT@ModernBERT
Max. piezo. (${e}_{ij}$)	LLM-Prop-35M	0.156	+4.5%	0.1490 ± 0.0026	imKT@BERT
Max. EFG	MatBERT-109M	26.621	+12.5%	23.30 ± 0.11	imKT@ModernBERT
Exfoliation energy	MatBERT-109M	37.445	+21.2%	29.5 ± 1.4	imKT@RoFormer
avg. ${m}_{e}$	MatBERT-109M	0.103	+18.7%	0.0837 ± 0.0010	imKT@ModernBERT
n-Seebeck	MatBERT-109M	58.342	+16.7%	48.6 ± 0.5	imKT@ModernBERT
n-PF	MatBERT-109M	528.070	+6.5%	493.7 ± 1.7	imKT@ModernBERT
p-Seebeck	MatBERT-109M	61.085	+17.8%	50.22 ± 0.06	imKT@ModernBERT
p-PF	LLM-Prop-35M	544.737	+12.2%	478.5 ± 1.4	imKT@ModernBERT
Band gap GGA	MatBERT-109M	0.461	+19.9%	0.3694 ± 0.0009	imKT@ModernBERT
Band gap HSE	MatBERT-109M	0.553	+21.5%	0.4341 ± 0.0027	imKT@ModernBERT
Band gap GGA optical	MatBERT-109M	0.701	+10.3%	0.629 ± 0.004	imKT@ModernBERT
Band gap HSE optical	MatBERT-109M	0.749	+9.1%	0.6811 ± 0.0015	imKT@ModernBERT
Castelli perovskites	AtomSets	0.082 ± 0.001	–	0.149 ± 0.010	imKT@RoFormer
Refractive index	Roost-SSL	0.3122 ± 0.0808	–	0.35 ± 0.09	imKT@RoFormer
shear modulus	CrabNet	0.092	+4.8%	0.0876 ± 0.0020	imKT@ModernBERT
bulk modulus	CrabNet	0.068	+1.6%	0.0669 ± 0.0031	imKT@ModernBERT
Experimental band gap	CrabNet	0.338	+7.7%	0.312 ± 0.022	imKT@RoFormer
MP formation energy	CrabNet	77	–	78.9 ± 1.7	imKT@ModernBERT
MP band gap	Finder	0.231	–	0.253 ± 0.004	imKT@RoFormer
Phonon peak	Roost-SSL	46.05 ± 4.22	–	54 ± 4	imKT@RoFormer

## Discussion

The presented approach—cross-modal knowledge transfer—is proved to be an effective route for enhancing efficacy of composition-based materials property prediction. Its modular structure (in both formulations) allows replacing distinct components. In the case of implicit transfer, we expect further enhancement owing to the development of multimodal representation learning and CLMs. On the other hand, explicit transfer can be advanced by resolving issues related to generating unplausible crystal structures; introducing multimodal foundational models as property predictors operating on hypothetical structures is another avenue for empowering the exKT pipeline. Finally, advanced ensembling techniques, e.g., mixture of experts, were shown to be a powerful auxiliary tool for increasing accuracy of heterogenous property predictors^34^; two formulations of cross-modal knowledge transfer can be unified within this approach.

## Methods

### Chemical language models (masked language modeling)

For modeling within chemical language models (CLMs), stoichiometric formulas of inorganic compounds, represented as sequences of atomic symbols and stoichiometric coefficients, were tokenized, e.g., [Na][1][Cl][1], [Li][1][Fe][1][Br][4], and [K][2][Si][4][O][9]; the chemical elements were arranged in order of increasing electronegativity (in accordance with the Ghosh scale^35^). At the first pretraining stage, masked language modeling^36,37^ (MLM) was carried out on preprocessed formulas. Cross-entropy ${\mathcal {L}}_{CE}$ was used for unmasking elemental symbols, whereas additional term, Number Token Loss^38^ (NTL) ${\mathcal {L}}_{NTL-WAS}$, was implemented to minimize the Wasserstein distance between the numerical values of the real and predicted number tokens. Finally, we prioritized thermodynamically stable compositions by introducing Boltzmann weights for training examples, taking into account an energy above the convex hull ${E}_{hull}$. The loss function value ${\mathcal {L}}^{(i)}$ for *i*-th example was computed as follows:

1 $$\begin{aligned} {\mathcal {L}}^{(i)} = \left( {\mathcal {L}}^{(i)}_{{CE}} + \lambda {\mathcal {L}}^{(i)}_{{NTL-WAS}} \right) \times \frac{{e}^{-{E}^{(i)}_{hull}}}{\sum _{j} {e}^{-{E}^{(j)}_{hull}}} \end{aligned}$$

where $\lambda$ is a normalized factor set to 0.5; Boltzmann summation was performed over a batch. Dataset consisting of the preprocessed formulas and corresponding formation energies (calculated at the generalized gradient approximation level of theory) was created by accumulating information from several density functional theory (DFT) databases: the Materials Project^39^ (MP), Open Quantum Materials Database^40^ (OQMD), Wang–Botti–Marques^41^ (WBM) dataset, and AFLOW database^42^. Considering the most stable polymorphs (i.e., with the lowest formation energy), energies above the convex hull were computed within the Quickhull algorithm^43^; the PatchedPhaseDiagram class (from the pymatgen.analysis.phase_diagram module) was utilized to partition high-dimensional chemical systems into low-dimensional chemical systems. The resulting dataset contained 952,792 entries, with the following contributions from the aforementioned DFT databases: 77,784 (MP), 487,855 (OQMD), 110,632 (WBM), and 276,521 (AFLOW). A training-validation-test ratio of 80:10:10 was utilized for the MLM task.

Three encoder-only transformer^22^ modifications were utilized: Bidirectional Encoder Representations from Transformers (BERT)^37^, enhanced transformer with rotary position embedding^44^ (RoFormer), and modernized encoder-only transformer^45^ (ModernBERT). All models, as implemented in the Hugging Face suite of libraries^46^, were trained for five epochs with the AdamW optimizer^47^ (a learning rate of ${10}^{-4}$ and a warmup ratio of 0.05), whereas the batch size was set to 256.

### Chemical language models (multimodal learning)

At the second pretraining stage, CLM outputs were aligned to multimodal representations. Inspiring by the multitask regression (MTR) architecture^48^, we pretrained BERT, RoFormer, and ModernBERT models on 128-dimensional embeddings taken from the crystal structure encoder (PotNet model^49^), which was implemented under the multimodal learning for materials (MultiMat) framework^8^. Specifically, the contrastive language image pre-training (CLIP) procedure^50^ was adopted in the original study to the materials domain: the AnchoredCLIP objective was proposed to align pairs consisting of the anchor modality (i.e., crystal structure) and each of the other modalities (i.e., density of states, charge density, and textual description).

Training, validation, and test subsets for MTR modeling included 125,753, 10,000 and 9,576 PotNet embeddings generated for the Materials Project crystal structures. CLMs were trained for 100 epochs with the AdamW optimizer^47^ (a learning rate of ${3 \times 10}^{-5}$ and a warmup ratio of 0.1); the batch size was set to 128.

### Chemical language models (downstream training)

Predictive performance of CLMs was assessed on several regression tasks taken from two well-established benchmarks. First, we benchmarked our models on eight MatBench endpoints^30^. Second, two compact (yet representative) datasets included in LLM4Mat-Bench^28^—JARVIS-DFT and SNUMAT—were examined as well. Although LLM4Mat-Bench features three input modalities of different granularity, including chemical composition, crystal text description, and Crystallographic Information File^51^ (CIF), only the former representation was considered. For all datasets, we utilized original training, validation, and test subsets to ensure compatibility with existing models. All CLMs were trained for 30 epochs with the AdamW optimizer^47^, a learning rate of ${3 \times 10}^{-5}$, and a warmup ratio of 0.1; the batch size was set to 64. To assess the robustness of the results with respect to stochastic initialization, three independent training runs were executed with distinct random seeds. Confidence intervals for the quality metrics, including MAE and RMSE, were evaluated based on the standard deviation across these three runs.

In addition to conventional mean absolute error (MAE), we computed the weighted average of MAD:MAE ratio^28^ as follows:

2 $$\begin{aligned} wtd. avg. (MAD:MAE) = \frac{\sum _{i}^{m} {TestSize}_{i} \times \frac{{MAD}_{i}}{{MAE}_{i}} }{\sum _{i}^{m} {TestSize}_{i}} \end{aligned}$$

where MAD is the mean absolute deviation; summation was performed over seven MatBench tasks (Supplementary Tables S4 and S5) except for experimental band gap.

### Explicit transfer from composition to structure domain

Materials property prediction based on chemical composition is fundamentally complicated by crystal polymorphism, i.e., existence of multiple crystalline phases of the same stoichiometry. Therefore, predictive models are forced to implicitly learn relationship between composition and target property value that correspond to specific (yet a priori unknown) crystal structure. Taking the reasonable as-sumption that the training set includes thermodynamically accessible compounds, the problem can be reformulated as the two-stage procedure, including the transfer from compositional to crystalline do-main, i.e., crystal structure prediction (CSP), and materials property prediction based on the generated crystal structures.

CSP is typically addressed by leveraging optimization algorithms and potential energy estimators (e.g., density functional theory and machine learning interatomic potentials). Taking into account the number of chemical systems under consideration (thousands of distinct compositions), our options are limited to approximate high-throughput approaches, opposing ab initio techniques. In this regard, we utilized a transformer-based, decoder-only language model, CrystaLLM, aimed at generating the crystal structure as a sequence of tokens, producing CIF files. The model was conditioned by a prompt containing two substrings: the data_ symbol and chemical formula with elements sorted by electronegativity (in accordance with the Pauling scale) and explicit stoichiometric indices; e.g., data_Na1Cl1, data_Li1Fe1Br4, and data_K2Si4O9. The model performed five attempts to generate a structure, using top-k sampling with *k* of 10 and a temperature of 1.0. For further analysis, the generated CIF files (read with the ase.io.read routine) were converted into the pymatgen.core.structure.Structure instances with pymatgen.io.ase.AseAtomsAdaptor and rewritten. The structures that failed the aforementioned conversion were eliminated. To quantify the extent of possible survivorship bias introduced by this filtering step, we additionally monitored the validity of the generated CIF files. Considering up to five generation attempts per composition, the fraction of valid generated structures retained for downstream analysis was 99.90% for SNUMAT, 99.93% for JARVIS-DFT, and 97.79% for the combined MatBench dataset considered in this work. In the case of MatBench, 13,077 generated CIF files failed pymatgen parsing and were discarded. Most compositions yielded five valid generated structures, namely 10,322/10,372 for SNUMAT, 75,746/75,965 for JARVIS-DFT, and 112,346/118,411 for MatBench. For compositions with fewer than five valid generated structures, predictions were averaged over the available valid structures only.

### Training of graph neural networks

CIF files generated in the previous step were utilized to train structure-aware predictive models, i.e., Graph Neural Networks (GNNs). We considered three GNN architectures: Crystal Graph Convolutional Neural Network^4^ (CGCNN), MatErials Graph Network^5^ (MEGNet), and Cartesian encoding graph neural Network^7^ (CartNet). Composition-wise predictions were produced by averaging model outputs corresponding to generated structures (up to five per composition). All models were optimized using the AdamW optimizer. CGCNN was trained with a learning rate of ${2 \times 10}^{-3}$ and batch size of 100; the number of epochs was set to 250. MEGNet was trained with a learning rate of ${5 \times 10}^{-4}$ and batch size of 100; the number of epochs was set to 250. CartNet was trained with a learning rate of ${10}^{-3}$ and batch size of 64; the number of epochs was set to 500. To ensure direct comparability with the CLM-based models, the exKT models were trained and evaluated using the same predefined training, validation, and test splits provided by the benchmark datasets. For each GNN architecture, three independent runs were carried out with distinct random seeds. All reported exKT metrics are presented as mean values with standard deviations across these runs. In addition, the set of generated CIF files was fixed before downstream GNN training and reused in all runs.

## Supplementary Information

Supplementary Information.

## Data Availability

The datasets analyzed during the current study are available in the following repositories: https://github.com/vertaix/LLM4Mat-Bench (LLM4Mat-Bench dataset), https://github.com/anthony-wang/CrabNet (MatBench dataset). The source code for preprocessing data and training models is available in the Multimat-ModernBERT repository, https://github.com/korolewadim/multimat-modernbert. The ModernBERT-base model pretrained via a two-step procedure is available as the HuggingFace model at https://huggingface.co/korolewadim/multimat-modernbert.
