# Supplementary material for: Enhancing composition-based materials property prediction by cross-modal knowledge transfer
Source: Sci Rep. 2026 May 20;16:16434. doi: 10.1038/s41598-026-53182-3 (PMC13216519; doi:10.1038/s41598-026-53182-3)
Supplement: Supplementary file 1 — Supplementary Information. [file 41598_2026_53182_MOESM1_ESM.pdf]

Supplementary material

Enhancing composition-based materials property prediction by cross-modal knowledge transfer

Ivan Rubtsov[1,2], *Ivan Dudakov*[1,2], *Yuri Kuratov*[3,2], *and Vadim Korolev*[1,2,*]

[1]AI Center, Lomonosov Moscow State University, Moscow, 119991, Russia
[2]MSU Institute for Artificial Intelligence, Lomonosov Moscow State University, Moscow, 119192, Russia
[3]Cognitive AI Systems Lab, Moscow, Russia

* korolevvv_01@my.msu.ru (Vadim Korolev): the author to whom correspondence should be addressed

Table S1. Predictive performance comparison of transfer knowledge models with existing architectures (LLM4Mat-Bench, JARVIS-DFT dataset). The metrics are provided in accordance with ref.[1]

	FEPA	Band gap (OPT)	Total energy	Ehull	Band gap (MBJ)
Gemma2-9b-it:5S	5.736	87.117	90.989	0.445	1.894
MatBERT-109M	0.126	0.235	0.194	**0.096**	0.491
LLM-Prop-35M	0.180	0.366	0.301	0.129	0.631
imKT@BERT	0.1352 ± 0.0018	**0.1985 ± 0.0019**	0.188 ± 0.008	0.1034 ± 0.0010	0.3876 ± 0.0029
imKT@RoFormer	0.1269 ± 0.0007	0.1993 ± 0.0027	0.1537 ± 0.0025	0.1031 ± 0.0009	0.3782 ± 0.0032
imKT@ModernBERT	**0.11488 ± 0.00018**	0.1994 ± 0.0019	**0.1172 ± 0.0005**	0.10486 ± 0.00011	**0.3773 ± 0.0030**
exKT@CGCNN	0.1267 ± 0.0005	0.2076 ± 0.0024	0.1362 ± 0.0008	0.11031 ± 0.00026	0.431 ± 0.005
exKT@MEGNet	0.1236 ± 0.0006	0.2105 ± 0.0024	0.1349 ± 0.0017	0.1068 ± 0.0011	0.419 ± 0.007
exKT@CartNet	0.1294 ± 0.0006	0.2132 ± 0.0024	0.1366 ± 0.0012	0.1180 ± 0.0013	0.413 ± 0.004

	Kv	Gv	SLME	Spillage	ϵ_x (OPT)
Gemma2-9b-it:5S	42.779	24.141	15.256	1.949	56.098
MatBERT-109M	18.498	14.241	5.851	0.409	32.661
LLM-Prop-35M	24.868	16.603	6.689	0.446	37.993
imKT@BERT	16.72 ± 0.11	13.24 ± 0.18	5.14 ± 0.10	0.356 ± 0.006	24.80 ± 0.22
imKT@RoFormer	16.72 ± 0.17	13.21 ± 0.12	5.00 ± 0.05	0.348 ± 0.006	24.65 ± 0.09
imKT@ModernBERT	**16.35 ± 0.24**	**12.76 ± 0.05**	**4.911 ± 0.010**	**0.3462 ± 0.0029**	**24.32 ± 0.06**
exKT@CGCNN	17.57 ± 0.23	13.73 ± 0.11	5.50 ± 0.14	0.379 ± 0.004	25.8 ± 0.4
exKT@MEGNet	17.05 ± 0.23	13.98 ± 0.06	5.50 ± 0.10	0.381 ± 0.007	25.367 ± 0.032
exKT@CartNet	17.24 ± 0.22	14.70 ± 0.13	5.179 ± 0.035	0.384 ± 0.004	26.54 ± 0.11

Table S2. Predictive performance comparison of transfer knowledge models with existing architectures (LLM4Mat-Bench, JARVIS-DFT dataset). The metrics are provided in accordance with ref.[1]

	ϵ (DFPT)	Max. piezo. (dij)	Max. piezo. (eij)	Max. EFG	Exfoliation energy
Gemma2-9b-it:5S	28.228	**7.973**	0.289	66.135	63.957
MatBERT-109M	28.340	9.920	0.172	26.621	37.445
LLM-Prop-35M	29.881	10.037	0.156	31.966	40.385
imKT@BERT	26.6 ± 0.7	10.21 ± 0.25	**0.1490 ± 0.0026**	24.4 ± 0.5	31.5 ± 1.5
imKT@RoFormer	**26.6 ± 0.4**	9.80 ± 0.27	0.1491 ± 0.0023	23.8 ± 0.5	**29.5 ± 1.4**
imKT@ModernBERT	28.3 ± 0.9	9.67 ± 0.10	0.1524 ± 0.0010	**23.30 ± 0.11**	30.26 ± 0.30
exKT@CGCNN	33 ± 5	9.68 ± 0.22	0.160 ± 0.005	26.6 ± 0.6	40 ± 6
exKT@MEGNet	27.1 ± 0.8	9.67 ± 0.14	0.1558 ± 0.0020	26.8 ± 0.8	41 ± 4
exKT@CartNet	27.4 ± 0.4	10.51 ± 0.13	0.177 ± 0.006	26.44 ± 0.19	29.67 ± 0.34

	avg. m_e	n-Seebeck	n-PF	p-Seebeck	p-PF
Gemma2-9b-it:5S	1.808	121.948	1209.64	182.513	890.704
MatBERT-109M	0.103	58.342	528.070	61.085	890.704
LLM-Prop-35M	0.130	64.538	608.007	75.268	544.737
imKT@BERT	**0.0837 ± 0.0010**	48.83 ± 0.12	515.6 ± 2.6	52.4 ± 0.5	498 ± 5
imKT@RoFormer	0.0865 ± 0.0024	48.8 ± 0.4	510.1 ± 3.2	51.4 ± 0.8	496 ± 9
imKT@ModernBERT	**0.0837 ± 0.0009**	**48.6 ± 0.5**	**493.7 ± 1.7**	**50.22 ± 0.06**	**478.5 ± 1.4**
exKT@CGCNN	0.0992 ± 0.0022	52.93 ± 0.21	537.7 ± 0.7	55.8 ± 0.6	530 ± 5
exKT@MEGNet	0.0957 ± 0.0008	52.35 ± 0.31	544 ± 7	56.6 ± 0.6	538 ± 5
exKT@CartNet	0.113 ± 0.009	53.4 ± 1.1	512 ± 8	55.9 ± 0.6	519 ± 6

Table S3. Predictive performance comparison of transfer knowledge models with existing architectures (LLM4Mat-Bench, SNUMAT dataset). The metrics are provided in accordance with ref.[1]

	Band gap GGA	Band gap HSE	Bandgap GGA optical	Bandgap HSE optical
Gemma2-9b-it:5S	1.182	0.935	1.876	1.585
MatBERT-109M	0.461	0.553	0.701	0.749
LLM-Prop-35M	0.571	0.674	0.829	0.901
imKT@BERT	0.3893 ± 0.0017	0.4441 ± 0.0016	0.643 ± 0.007	0.697 ± 0.005
imKT@RoFormer	0.379 ± 0.005	0.436 ± 0.006	0.650 ± 0.006	0.713 ± 0.006
imKT@ModernBERT	**0.3694 ± 0.0009**	**0.4341 ± 0.0027**	**0.629 ± 0.004**	**0.6811 ± 0.0015**
exKT@CGCNN	0.430 ± 0.005	0.482 ± 0.007	0.703 ± 0.009	0.7514 ± 0.0010
exKT@MEGNet	0.4019 ± 0.0010	0.465 ± 0.008	0.694 ± 0.011	0.746 ± 0.013
exKT@CartNet	0.3860 ± 0.0011	0.4526 ± 0.0014	0.679 ± 0.006	0.718 ± 0.004

Table S4. Predictive performance comparison of transfer knowledge models with existing architectures (MatBench tasks). The metrics are provided in accordance with the outlined references.

	Castelli perovskites	Refractive index	log_{10}(shear modulus)	log_{10}(bulk modulus)
ElemNet[2]	0.194	0.442	0.125	0.090
CrabNet[2]	0.127	0.348	0.092	0.068
HotCrab[2]	0.135	0.366	0.097	0.072
Roost[3]	0.4025 ± 0.0077	0.3252 ± 0.0780	0.1034 ± 0.0020	0.0797 ± 0.0042
Roost-SSL[3]	0.4050 ± 0.0086	**0.3122 ± 0.0808**	0.1006 ± 0.0023	0.0777 ± 0.0041
Roost-FL[3]	0.4043 ± 0.0091	0.3167 ± 0.0779	0.1046 ± 0.0029	0.0776 ± 0.0031
Roost-ML[3]	0.4013 ± 0.0077	0.3221 ± 0.0761	0.1032 ± 0.0021	0.0782 ± 0.0047
Finder[3]	0.645	0.3204	0.0996	0.0764
AtomSets[4]	**0.082 ± 0.001**	0.36 ± 0.07	0.11 ± 0.00	0.08 ± 0.00
AtomSets-V_0[4]	**0.082 ± 0.002**	0.35 ± 0.08	0.11 ± 0.00	0.08 ± 0.00
imKT@BERT	0.154 ± 0.007	0.35 ± 0.10	0.0909 ± 0.0033	0.0694 ± 0.0033
imKT@RoFormer	0.149 ± 0.010	0.35 ± 0.09	0.0878 ± 0.0010	0.0675 ± 0.0031
imKT@ModernBERT	0.152 ± 0.004	0.36 ± 0.09	**0.0876 ± 0.0020**	**0.0669 ± 0.0031**
exKT@CGCNN	0.158 ± 0.006	0.43 ± 0.10	0.1096 ± 0.0035	0.085 ± 0.004
exKT@MEGNet	0.161 ± 0.007	0.46 ± 0.10	0.1039 ± 0.0032	0.083 ± 0.005
exKT@CartNet	0.158 ± 0.005	0.41 ± 0.09	0.108 ± 0.014†	0.0802 ± 0.0034‡

† The metric is provided for the subset excluding outliers in predictions (one predicted value exceeded the ground truth by more than ten times).
‡ The metric is provided for the subset excluding outliers in predictions (one predicted value exceeded the ground truth by more than ten times).

Table S5. Predictive performance comparison of transfer knowledge models with existing architectures (MatBench tasks). The metrics are provided in accordance with the outlined references.

	Exp. band gap	MP formation energy	MP band gap	Phonon peak	Wtd. Avg. ↑
ElemNet[2]	0.439	746	0.313	–	–
CrabNet[2]	0.338	**77**	0.263	53.341	**8.25**
HotCrab[2]	0.352	80	0.273	60.253	7.93
Roost[3]	–	84.7 ± 1.6	0.2571 ± 0.0055	54.38 ± 4.73	7.69
Roost-SSL[3]	–	85.4 ± 1.2	0.2646 ± 0.0041	**46.05 ± 4.22**	7.61
Roost-FL[3]	–	84.3 ± 1.7	0.2560 ± 0.0037	51.93 ± 6.95	7.73
Roost-ML[3]	–	83.4 ± 1.0	0.2551 ± 0.0104	53.33 ± 5.61	7.80
Finder[3]	–	83.9	**0.231**	46.6	7.98
AtomSets[4]	0.43 ± 0.03	94 ± 1	0.26 ± 0.01	63 ± 12	7.18
AtomSets-V_0[4]	0.41 ± 0.03	95 ± 1	0.26 ± 0.00	53 ± 15	7.14
imKT@BERT	0.329 ± 0.026	94.9 ± 1.1	0.2542 ± 0.0028	55 ± 4	7.21
imKT@RoFormer	**0.312 ± 0.022**	85.9 ± 1.1	0.253 ± 0.004	54 ± 4	7.73
imKT@ModernBERT	0.348 ± 0.020	78.9 ± 1.7	0.263 ± 0.004	56 ± 4	8.11
exKT@CGCNN	–	112 ± 4	0.307 ± 0.007	76 ± 8	6.06
exKT@MEGNet	–	106.4 ± 2.6	0.294 ± 0.004	77 ± 10	6.35
exKT@CartNet	–	103.4 ± 1.2[§]	0.305 ± 0.009[**]	71 ± 10	6.42

[§] The metric is provided for the subset excluding outliers in predictions (nine predicted values exceeded the ground truth by more than ten times).
[**] The metric is provided for the subset excluding outliers in predictions (seven predicted values exceeded the ground truth by more than ten times).

Table S6. Predictive performance comparison of implicit transfer knowledge models (LLM4Mat-Bench, JARVIS-DFT dataset). Non-pretrained models are compared with three pretraining strategies: masked language modeling (MLM), multimodal learning (MML), and their combination.

	FEPA	Band gap (OPT)	Total energy	Ehull	Band gap (MBJ)
BERT from scratch	0.13328 ± 0.00032	0.2617 ± 0.0007	0.160 ± 0.006	0.1103 ± 0.0010	0.472 ± 0.008
BERT MLM	0.1284 ± 0.0007	0.2113 ± 0.0011	**0.14915 ± 0.00030**	0.10403 ± 0.00032	0.413 ± 0.011
BERT MML	**0.1253 ± 0.0008**	**0.1917 ± 0.0013**	0.1612 ± 0.0013	**0.1026 ± 0.0009**	**0.3862 ± 0.0034**
BERT MLM+MML	0.1352 ± 0.0018	0.1985 ± 0.0019	0.188 ± 0.008	0.1034 ± 0.0010	0.3876 ± 0.0029
RoFormer from scratch	0.1251 ± 0.0006	0.268 ± 0.009	**0.1414 ± 0.0004**	0.1076 ± 0.0009	0.497 ± 0.008
RoFormer MLM	0.1319 ± 0.0010	0.2139 ± 0.0031	0.160 ± 0.004	0.1043 ± 0.0008	0.4104 ± 0.0025
RoFormer MML	**0.1217 ± 0.0011**	**0.1985 ± 0.0018**	0.1488 ± 0.0014	**0.1027 ± 0.0006**	0.390 ± 0.007
RoFormer MLM+MML	0.1269 ± 0.0007	0.1993 ± 0.0027	0.1537 ± 0.0025	0.1031 ± 0.0009	**0.3782 ± 0.0032**
ModernBERT from scratch	0.11763 ± 0.00017	0.2750 ± 0.0020	0.1253 ± 0.0020	0.1069 ± 0.0004	0.490 ± 0.011
ModernBERT MLM	0.11908 ± 0.00027	0.2090 ± 0.0013	0.1206 ± 0.0004	0.1061 ± 0.0004	0.4025 ± 0.0034
ModernBERT MML	**0.11171 ± 0.00035**	**0.1923 ± 0.0012**	**0.1166 ± 0.0005**	**0.10183 ± 0.00032**	**0.3766 ± 0.0021**
ModernBERT MLM+MML	0.11488 ± 0.00018	0.1994 ± 0.0019	0.1172 ± 0.0005	0.10486 ± 0.00011	0.3773 ± 0.0030

	Kv	Gv	SLME	Spillage	ϵ_x (OPT)
BERT from scratch	17.22 ± 0.11	14.10 ± 0.08	5.73 ± 0.09	0.392 ± 0.004	26.23 ± 0.18
BERT MLM	16.71 ± 0.14	13.70 ± 0.17	5.12 ± 0.04	0.3689 ± 0.0009	25.04 ± 0.17
BERT MML	**16.26 ± 0.21**	**12.873 ± 0.033**	**4.88 ± 0.05**	**0.349 ± 0.005**	**24.45 ± 0.12**
BERT MLM+MML	16.72 ± 0.11	13.24 ± 0.18	5.14 ± 0.10	0.356 ± 0.006	24.80 ± 0.22
RoFormer from scratch	17.022 ± 0.033	14.10 ± 0.15	5.70 ± 0.05	0.3823 ± 0.0012	25.60 ± 0.07
RoFormer MLM	16.84 ± 0.13	13.38 ± 0.04	5.09 ± 0.04	0.369 ± 0.005	25.13 ± 0.27
RoFormer MML	**16.23 ± 0.34**	**13.19 ± 0.10**	**5.00 ± 0.04**	**0.344 ± 0.005**	**24.52 ± 0.09**
RoFormer MLM+MML	16.72 ± 0.17	13.21 ± 0.12	**5.00 ± 0.05**	0.348 ± 0.006	24.65 ± 0.09
ModernBERT from scratch	17.03 ± 0.05	14.22 ± 0.05	5.51 ± 0.07	0.3823 ± 0.0030	26.19 ± 0.20
ModernBERT MLM	16.673 ± 0.019	13.427 ± 0.034	5.210 ± 0.026	0.364 ± 0.005	25.047 ± 0.031
ModernBERT MML	**16.26 ± 0.07**	12.97 ± 0.08	5.008 ± 0.030	0.35180 ± 0.00015	24.34 ± 0.06
ModernBERT MLM+MML	16.35 ± 0.24	**12.76 ± 0.05**	**4.911 ± 0.010**	**0.3462 ± 0.0029**	**24.32 ± 0.06**

Table S7. Predictive performance comparison of implicit transfer-knowledge models (LLM4Mat-Bench, JARVIS-DFT dataset). Non-pretrained models are compared with three pretraining strategies: masked language modeling (MLM), multimodal learning (MML), and their combination.

	ϵ (DFPT)	Max. piezo. (dij)	Max. piezo. (eij)	Max. EFG	Exfoliation energy
BERT from scratch	28.36 ± 0.20	10.57 ± 0.05	0.1690 ± 0.0025	28.97 ± 0.31	38.49 ± 0.16
BERT MLM	27.4 ± 0.5	**9.47 ± 0.26**	**0.1490 ± 0.0020**	25.82 ± 0.18	32.7 ± 0.4
BERT MML	27.2 ± 1.2	9.55 ± 0.19	0.1506 ± 0.0008	**23.55 ± 0.21**	**29.1 ± 1.4**
BERT MLM+MML	**26.6 ± 0.7**	10.21 ± 0.25	**0.1490 ± 0.0026**	24.4 ± 0.5	31.5 ± 1.5
RoFormer from scratch	27.98 ± 0.09	10.55 ± 0.12	0.17037 ± 0.00029	28.79 ± 0.35	38.2 ± 0.8
RoFormer MLM	27.4 ± 1.3	9.62 ± 0.17	0.1510 ± 0.0023	25.33 ± 0.14	34.0 ± 0.7
RoFormer MML	**26.2 ± 0.9**	**9.41 ± 0.10**	**0.1459 ± 0.0013**	23.89 ± 0.12	**27.7 ± 1.3**
RoFormer MLM+MML	26.6 ± 0.4	9.80 ± 0.27	0.1491 ± 0.0023	**23.8 ± 0.5**	29.5 ± 1.4
ModernBERT from scratch	28.43 ± 0.20	10.41 ± 0.07	0.1670 ± 0.0011	28.34 ± 0.27	37.7 ± 0.5
ModernBERT MLM	27.1 ± 0.5	9.6 ± 0.6	0.1528 ± 0.0021	24.99 ± 0.11	30.7 ± 0.6
ModernBERT MML	**26.16 ± 0.08**	**9.4 ± 0.4**	**0.1478 ± 0.0009**	23.72 ± 0.05	30.3 ± 0.4
ModernBERT MLM+MML	28.3 ± 0.9	9.67 ± 0.10	0.1524 ± 0.0010	**23.30 ± 0.11**	**30.26 ± 0.30**

	avg. m_e	n-Seebeck	n-PF	p-Seebeck	p-PF
BERT from scratch	0.1037 ± 0.0010	58.6 ± 0.6	543.3 ± 2.8	63.5 ± 1.0	527.8 ± 1.1
BERT MLM	0.0918 ± 0.0009	52.2 ± 0.6	518 ± 6	55.4 ± 0.6	511.6 ± 1.6
BERT MML	0.0849 ± 0.0007	**48.38 ± 0.33**	**506.4 ± 0.6**	**52.1 ± 0.4**	**493.1 ± 2.4**
BERT MLM+MML	**0.0837 ± 0.0010**	48.83 ± 0.12	515.6 ± 2.6	52.4 ± 0.5	498 ± 5
RoFormer from scratch	0.1027 ± 0.0031	59.56 ± 0.31	533.3 ± 2.3	65.0 ± 0.7	520.9 ± 2.4
RoFormer MLM	0.0945 ± 0.0031	52.7 ± 0.8	522 ± 7	55.4 ± 0.9	505 ± 8
RoFormer MML	**0.0857 ± 0.0013**	**48.2 ± 0.4**	**496.2 ± 2.3**	**51.4 ± 0.4**	**482.3 ± 1.7**
RoFormer MLM+MML	0.0865 ± 0.0024	48.8 ± 0.4	510.1 ± 3.2	**51.4 ± 0.8**	496 ± 9
ModernBERT from scratch	0.1061 ± 0.0023	58.12 ± 0.17	536.4 ± 3.0	62.5 ± 0.7	525.9 ± 1.6
ModernBERT MLM	0.0913 ± 0.0014	52.30 ± 0.05	515.2 ± 2.7	53.23 ± 0.32	500.7 ± 0.6
ModernBERT MML	0.0852 ± 0.0027	**48.3 ± 0.4**	502 ± 7	51.2 ± 0.7	488.5 ± 1.9
ModernBERT MLM+MML	**0.0837 ± 0.0009**	48.6 ± 0.5	**493.7 ± 1.7**	**50.22 ± 0.06**	**478.5 ± 1.4**

Table S8. Predictive performance comparison of implicit transfer-knowledge models (LLM4Mat-Bench, SNUMAT dataset). Non-pretrained models are compared with three pretraining strategies: masked language modeling (MLM), multimodal learning (MML), and their combination.

	Band gap GGA	Band gap HSE	Bandgap GGA optical	Bandgap HSE optical
BERT from scratch	0.487 ± 0.007	0.5633 ± 0.0015	0.762 ± 0.010	0.8093 ± 0.0015
BERT MLM	0.432 ± 0.007	0.495 ± 0.007	0.6795 ± 0.0016	0.766 ± 0.010
BERT MML	**0.3722 ± 0.0030**	**0.429 ± 0.006**	**0.632 ± 0.006**	0.6971 ± 0.0016
BERT MLM+MML	0.3893 ± 0.0017	0.4441 ± 0.0016	0.643 ± 0.007	**0.697 ± 0.005**
RoFormer from scratch	0.480 ± 0.004	0.5680 ± 0.0033	0.742 ± 0.009	0.819 ± 0.012
RoFormer MLM	0.4264 ± 0.0017	0.499 ± 0.004	0.701 ± 0.006	0.7677 ± 0.0010
RoFormer MML	**0.3700 ± 0.0026**	**0.425 ± 0.005**	**0.637 ± 0.008**	**0.685 ± 0.004**
RoFormer MLM+MML	0.379 ± 0.005	0.436 ± 0.006	0.650 ± 0.006	0.713 ± 0.006
ModernBERT from scratch	0.4954 ± 0.0029	0.572 ± 0.005	0.7570 ± 0.0030	0.816 ± 0.004
ModernBERT MLM	0.4175 ± 0.0034	0.4752 ± 0.0019	0.6579 ± 0.0027	0.7095 ± 0.0019
ModernBERT MML	**0.3634 ± 0.0011**	**0.4256 ± 0.0006**	0.62989 ± 0.00027	0.6850 ± 0.0035
ModernBERT MLM+MML	0.3694 ± 0.0009	0.4341 ± 0.0027	**0.629 ± 0.004**	**0.6811 ± 0.0015**

Table S9. Predictive performance comparison of implicit transfer knowledge models (MatBench tasks). Non-pretrained models are compared with three pretraining strategies: masked language modeling (MLM), multimodal learning (MML), and their combination.

	Castelli perovskites	Refractive index	$\log_{10}$(shear modulus)	$\log_{10}$(bulk modulus)
BERT from scratch	0.146 ± 0.004	0.43 ± 0.09	0.1167 ± 0.0021	0.0829 ± 0.0029
BERT MLM	0.151 ± 0.005	0.38 ± 0.10	0.1006 ± 0.0020	0.0749 ± 0.0034
BERT MML	**0.1454 ± 0.0025**	**0.34 ± 0.09**	**0.0886 ± 0.0021**	**0.0682 ± 0.0028**
BERT MLM+MML	0.154 ± 0.007	0.35 ± 0.10	0.0909 ± 0.0033	0.0694 ± 0.0033
RoFormer from scratch	0.139 ± 0.008	0.43 ± 0.09	0.1151 ± 0.0032	0.0812 ± 0.0032
RoFormer MLM	0.153 ± 0.005	0.38 ± 0.10	0.0988 ± 0.0021	0.0742 ± 0.0026
RoFormer MML	**0.148 ± 0.006**	0.36 ± 0.10	**0.0865 ± 0.0022**	0.0690 ± 0.0027
RoFormer MLM+MML	0.149 ± 0.010	**0.35 ± 0.09**	0.0878 ± 0.0010	**0.0675 ± 0.0031**
ModernBERT from scratch	**0.132 ± 0.006**	0.42 ± 0.09	0.1178 ± 0.0022	0.084 ± 0.004
ModernBERT MLM	0.157 ± 0.010	0.38 ± 0.10	0.1002 ± 0.0025	0.0757 ± 0.0034
ModernBERT MML	0.149 ± 0.010	**0.35 ± 0.09**	0.0881 ± 0.0022	0.0673 ± 0.0030
ModernBERT MLM+MML	0.152 ± 0.004	0.36 ± 0.09	**0.0876 ± 0.0020**	**0.0669 ± 0.0031**

Table S10. Predictive performance comparison of implicit transfer knowledge models (MatBench tasks). Non-pretrained models are compared with three pretraining strategies: masked language modeling (MLM), multimodal learning (MML), and their combination.

	Experimental band gap	MP formation energy	MP band gap	Phonon peak
BERT from scratch	0.477 ± 0.033	113.9 ± 1.0	0.3458 ± 0.0025	103 ± 11
BERT MLM	0.381 ± 0.020	**94.7 ± 2.1**	0.280 ± 0.004	62 ± 6
BERT MML	**0.328 ± 0.019**	95.6 ± 0.6	0.2627 ± 0.0019	**52 ± 4**
BERT MLM+MML	0.329 ± 0.026	94.9 ± 1.1	**0.2542 ± 0.0028**	55 ± 4
RoFormer from scratch	0.469 ± 0.030	107.6 ± 2.1	0.343 ± 0.004	110 ± 12
RoFormer MLM	0.368 ± 0.015	99.3 ± 1.9	0.2803 ± 0.0029	69 ± 4
RoFormer MML	0.327 ± 0.028	**85.8 ± 1.9**	0.2562 ± 0.0030	**52 ± 8**
RoFormer MLM+MML	**0.312 ± 0.022**	85.9 ± 1.1	**0.253 ± 0.004**	54 ± 4
ModernBERT from scratch	0.452 ± 0.020	102.8 ± 1.7	0.3399 ± 0.0035	93 ± 8
ModernBERT MLM	0.416 ± 0.017	89.5 ± 1.6	0.2862 ± 0.0013	65 ± 8
ModernBERT MML	**0.330 ± 0.019**	85.7 ± 1.3	**0.262 ± 0.004**	**52 ± 4**
ModernBERT MLM+MML	0.348 ± 0.020	**78.9 ± 1.7**	0.263 ± 0.004	56 ± 4

REFERENCES

1. Rubungo, A. N., Li, K., Hattrick-Simpers, J. & Dieng, A. B. LLM4Mat-bench: benchmarking large language models for materials property prediction. *Mach. Learn. Sci. Technol.* **6**, 20501 (2025).

2. Wang, A. Y.-T., Kauwe, S. K., Murdock, R. J. & Sparks, T. D. Compositionally restricted attention-based network for materials property predictions. *npj Comput. Mater.* **7**, 1–10 (2021).

3. Huang, H., Magar, R. & Barati Farimani, A. Pretraining strategies for structure agnostic material property prediction. *J. Chem. Inf. Model.* **64**, 627–637 (2024).

4. Chen, C. & Ong, S. P. AtomSets as a hierarchical transfer learning framework for small and large materials datasets. *npj Comput. Mater.* **7**, 173 (2021).
